# The SSRI Clinic: Improving SSRI Prescribing Safety in Outpatient CAMHS Clinic

**DOI:** 10.1192/bjo.2022.405

**Published:** 2022-06-20

**Authors:** Tammy Morgan, Helen McFerran, Anna McGovern

**Affiliations:** Southern Health and Social Care Trust, Belfast, United Kingdom

## Abstract

**Aims:**

Within a multidisciplinary team of medical and non medical prescribers the aim of this project was to improve SSRI prescribing safety by 30% by June 2022. This was with view to enhance prescribing provision across the trust.

**Methods:**

Multiple methods were done to improve staffs perception of safety. Criteria were set out in keeping with NICE guidance, RCPSYCH and BAP guidance on prescribing. Psychoeducation and focus groups were held to gauge colleagues thoughts on SSRI prescribing. This was along with pulse surveys.

An SSRI clinic was set up, with referral pathway, protocol for referral and staff clinics for reviews and new prescribing. This was to improve prescribing safety.

Health promotion leaflets were also made for the clinic in terms of non pharmacological methods to improve mental health.

**Results:**

Improved staff safety from a Good (3) to Excellent (5).

Established SSRI clinic which will be spread trust wide to the other clinics.

Better monitoring and education of SSRIs.

Health promotion benefits.

**Conclusion:**

Improved staff safety from a Good (3) to Excellent (5).

Established SSRI clinic which will be spread trust wide to the other clinics.

Better monitoring and education of SSRIs.

Health promotion benefits.

